# Supplementary material for: Diversity Patterns of Macrofungi in Xerothermic Grasslands from the Nida Basin (Małopolska Upland, Southern Poland): A Case Study
Source: Biology (Basel). 2022 Mar 30;11(4):531. doi: 10.3390/biology11040531 (PMC9028154; doi:10.3390/biology11040531)
Supplement: Supplementary file 1 [file biology-11-00531-s001.zip › Table S1.pdf]

Table S1: Geographical characteristics of sampling sites for the fungi in xerothermic grasslands from the Nida Basin (Małopolska Upland).

Association *Festucetum pallentis*

Locality	Latitude	Longitude	Exposition	Inclination	Cover %
Gacki	50.452929	20.586970	S	45	60
Wola Zagojska	50.438353	20.614052	S	40	50
Wola Zagojska	50.438291	20.614255	S	20	60
Wola Zagojska	50.438272	20.615538	W	45	60
Wola Zagojska	50.442806	20.610783	W	30	60

Association *Sisymbrio-Stipetum capillatae*

Locality	Latitude	Longitude	Exposition	Inclination	Cover %
Skorocice	50.424308	20.675142	S	2	90
Skorocice	50.424727	20.675221	S	2	85
Skorocice	50.425860	20.675144	S	3	95
Wola Zagojska	50.445159	20.608391	S	30	80
Wola Zagojska	50.442686	20.610943	SE	10	100

Association *Inuletum ensifoliae*

Locality	Latitude	Longitude	Exposition	Inclination	Cover %
Pińczów	50.535321	20.518402	S	25	100
Pińczów	50.535465	20.518682	S	30	100
Pińczów	50.535612	20.516728	S	5	100
Krzyżanowice	50.452717	20.560480	S	30	100
Gacki	50.452862	20.587532	SE	10	100

Association *Thalictro_Salvietum pratensis*

Locality	Latitude	Longitude	Exposition	Inclination	Cover %
Pińczów	50.535345	20.517840	S	20	60
Krzyżanowice	50.452561	20.559516	S	40	100
Krzyżanowice	50.452681	20.559752	S	20	60
Krzyżanowice	50.452817	20.559092	S	20	100
Gacki	50.452735	20.586702	S	2	100

Association *Adonido-Brachypodietum pinnati*

Locality	Latitude	Longitude	Exposition	Inclination	Cover %
Wola Zagojska	50.435532	20.619661	SWW	10	100
Wola Zagojska	50.435515	20.619841	SW	15	100

Wola Zagojska	50.435426	20.620066	SW	25	100
Krzyżanowice	50.453609	20.560148	SW	25	100
Skorocice	50.423894	20.674739	SEE	30	100

Association *Seslerio-Scorzoneretum purpureae*

Locality	Latitude	Longitude	Exposition	Inclination	Cover %
Wola Zagojska	50.435638	20.621039	E	15	100
Wola Zagojska	50.442784	20.610962	NNE	40	100
Krzyżanowice	50.454044	20.557868	N	30	100
Krzyżanowice	50.453961	20.558205	NE	20	100
Skorocice	50.423951	20.674558	N	35	100

Association *Koelerio-Festucetum rupicolae*

Locality	Latitude	Longitude	Exposition	Inclination	Cover %
Pińczów	50.535321	20.517840	SW	20	85
Skorocice	50.424727	20.675221	S	3	85
Wola Zagojska	50.264132	20.363461	SE	30	80
Krzyżanowice	50.271344	20.333668	N	30	100
Gacki	50.264162	20.363432	N	10	100
