# Supplementary material for: Diversity Patterns of Macrofungi in Xerothermic Grasslands from the Nida Basin (Małopolska Upland, Southern Poland): A Case Study
Source: Biology (Basel). 2022 Mar 30;11(4):531. doi: 10.3390/biology11040531 (PMC9028154; doi:10.3390/biology11040531)
Supplement: Supplementary file 1 [file biology-11-00531-s001.zip › Table S2.pdf]

Table S2: Results of the Monte Carlo permutation tests and forward selection performed during the CCA (canonical correspondence analysis)

Variable name	% of explained variation	Pseudo-F	*p*-value
TS (*Thalictro–Salvietum pratensis*)	7.3	1.9	**0.022**
Ie (*Innuletum ensifoliae*)	7.1	1.8	**0.044**
SSc (*Seslerio–Scorzoneretum purpureae*)	5.6	1.4	0.07
AB (*Adonido–Brachypodietum pinnati*)	4.7	1.2	0.24
KF (*Koelerio–Festucetum rupicole*)	3.9	1.0	0.448
Fp (*Festucetum pallentis*)	3.4	0.8	0.698
SSt (*Sisymbrio–Stipetum capillatae*)	2.9	0.7	0.886
