# Supplementary material for: Diversity Patterns of Macrofungi in Xerothermic Grasslands from the Nida Basin (Małopolska Upland, Southern Poland): A Case Study
Source: Biology (Basel). 2022 Mar 30;11(4):531. doi: 10.3390/biology11040531 (PMC9028154; doi:10.3390/biology11040531)
Supplement: Supplementary file 1 [file biology-11-00531-s001.zip › Table S3.pdf]

Table S3. Incidence of macrofungi fruiting bodies in the studied plant associations and years. Legend: **AB** *Adonido–Brachypodietum pinnati;* **Fp** *Festucetum pallentis;* **Ie** *Inuletum ensifoliae;* **KF** *Koelerio-Festucetum;* **SSc** *Seslerio–Scorzoneretum purpureae;* **SSt** *Sisymbrio–Stipetum capillatae;* **TS** *Thalictro-Salvietum pratensis.*

**year**	2010	2011	2012	2013	2010	2011	2012	2013	2010	2011	2012	2013	2010	2011	2012	2013	2010	2011	2012	2013	2010	2011	2012	2013	2010	2011	2012	2013
**association** / **species**	**Fp**	**Fp**	**Fp**	**Fp**	**SSt**	**SSt**	**SSt**	**SSt**	**KF**	**KF**	**KF**	**KF**	**AB**	**AB**	**AB**	**AB**	**Ie**	**Ie**	**Ie**	**Ie**	**SSc**	**SSc**	**SSc**	**SSc**	**TS**	**TS**	**TS**	**TS**
Agaricus arvensis Schaeff.	-	-	-	-	-	-	-	-	-	-	-	-	-	-	3	-	-	-	-	-	-	-	-	-	-	-	-	-
Agaricus bisporus (J. E. Lange) Imbach	-	-	-	-	-	-	-	-	-	-	-	-	-	-	3	-	-	-	-	-	-	-	-	-	-	-	-	-
Agaricus campestris L.	-	-	1	-	-	-	5	-	-	-	-	-	-	-	-	-	-	-	-	-	-	-	-	-	-	-	-	-
Agaricus xanthodermus Genev.	-	-	-	-	-	-	-	-	-	-	-	-	-	-	-	2	-	-	-	-	-	-	-	-	-	-	-	-
Agrocybe pediades (Fr.) Fayod	-	-	-	-	-	-	4	-	-	-	-	-	-	-	-	-	-	-	-	-	-	7	-	-	-	-	-	-
Aleuria aurantia (Pers.) Fuckel	-	-	-	-	-	-	-	-	-	-	-	-	-	-	-	-	-	-	-	-	5	-	-	-	-	-	-	-
Arrhenia griseopallida (Desm.) Watling	-	-	-	12	3	-	-	-	-	-	-	-	-	-	-	-	-	-	-	-	-	-	-	-	-	-	-	-
Atheniella delectabilis (Peck) Lüderitz & H. Lehmann	-	-	-	-	-	-	-	-	-	-	-	-	-	-	4	-	-	-	-	-	-	-	-	-	-	-	-	-
Atheniella flavoalba (Fr.) Redhead, Moncalvo, Vilgalys, Desjardin & B.A. Perry	-	-	-	19	-	-	20	-	-	-	5	-	-	-	44	-	-	-	-	-	-	-	-	-	-	-	27	-
Bovista aestivalis (Bonord.) Demoulin	-	2	2	-	-	-	-	-	-	-	-	-	-	-	-	-	-	-	-	-	-	-	-	-	-	-	-	-
Bovista limosa Rostr.	-	3	-	-	-	-	-	-	-	-	-	-	-	-	-	-	-	-	-	-	-	-	-	-	-	-	-	-
Bovista nigrescens Pers.	-	-	-	-	-	-	-	-	-	-	-	-	-	-	-	-	-	-	-	-	1	-	-	-	-	-	-	-
Bovista plumbea Pers.	-	1	1	-	-	1	-	-	-	-	-	-	1	-	-	-	-	-	-	-	-	-	-	-	-	-	-	-
Bovista tomentosa (Vittad.) De Toni	-	2	3	4	-	5	10	2	-	-	-	-	-	-	-	-	-	-	-	-	-	-	-	-	-	-	-	-
Bovistella utriformis (Bull.) Demoulin & Rebriev	-	-	-	-	3	-	-	-	-	-	-	-	-	-	-	-	-	-	-	-	-	-	-	-	-	-	-	-
Calvatia gigantea (Batsch) Lloyd	-	-	-	-	-	-	-	-	-	-	-	-	-	-	-	-	-	-	-	-	-	-	-	-	-	-	-	2
Calycina herbarum (Pers.) Gray	-	-	-	-	-	-	-	-	-	-	-	-	-	-	-	-	-	-	-	-	-	-	-	-	-	30	173	-

Chlorophyllum rhacodes (Vittad.) Vellinga	-	-	-	-	-	-	-	-	-	-	-	-	-	-	9	-	-	-	-	-	-	-	-	-	-	-	-	-
Clitocella popinalis (Fr.) Kluting, T.J. Baroni & Bergemann	-	-	1	1	-	-	-	-	-	-	-	-	-	-	-	-	-	-	-	-	-	-	-	-	-	-	-	-
Clitocybe agrestis Harmaja	-	-	-	-	-	-	6	3	-	-	-	-	-	-	-	-	-	-	-	-	-	-	-	-	-	-	-	-
Clitocybe dealbata (Sowerby) P. Kumm.	-	-	-	-	-	-	-	-	-	-	-	-	1	-	-	-	2	-	-	-	-	-	-	-	-	-	-	-
Conocybe apala (Fr.) Arnolds	-	-	-	-	3	-	-	-	-	-	-	-	-	-	-	-	-	-	-	-	-	-	-	-	-	-	-	-
Conocybe pseudocrispa (Hauskn.) Arnolds	-	-	2	-	-	-	-	-	-	-	-	-	-	-	-	-	-	-	-	-	-	-	-	-	-	-	-	-
Conocybe pubescens (Gillet) Kühner	-	-	-	-	-	-	-	-	-	-	-	-	1	-	-	-	-	-	-	-	-	-	-	-	-	-	-	-
Conocybe siennophylla (Berk. & Broome) Singer ex Chiari & Papetti	-	-	-	-	-	-	-	-	-	-	-	-	-	1	-	-	-	-	-	-	-	-	-	-	-	-	-	-
Conocybe tenera (Schaeff.) Kühner	-	-	-	-	-	-	-	-	-	-	-	-	-	1	-	-	-	-	-	-	-	-	-	-	-	-	-	-
Coprinopsis friesii (Quél.) P. Karst.	-	-	-	-	-	-	-	-	-	-	-	-	-	-	7	-	-	-	-	-	-	-	-	-	-	-	3	-
Coriolopsis gallica (Fr.) Ryvarden	-	-	-	-	-	-	-	-	23	-	-	-	-	-	-	-	-	-	-	-	-	-	-	-	-	-	-	-
Crepidotus epibryus (Fr.) Quél.	-	-	-	-	-	-	-	-	-	-	-	-	-	4	-	-	-	-	-	-	-	-	-	-	-	-	-	-
Crinipellis scabella (Alb. & Schwein.) Murrill	19	48	173	6	12	42	141	55	15	-	10	-	13	-	85	-	-	-	13	-	21	14	8	-	-	-	-	-
Crucibulum laeve (Huds.) Kambly	-	-	-	-	-	6	-	-	-	-	-	-	-	-	-	-	-	-	-	-	-	-	-	-	-	-	-	-
Cuphophyllus pratensis (Pers.) Bon	-	-	-	-	-	1	3	2	-	-	-	-	-	-	-	-	-	-	-	-	-	-	-	-	-	-	-	-
Cuphophyllus russocoriaceus (Berk. & T.K. Mill.) Bon	-	-	-	9	1	13	-	-	-	-	-	-	-	-	-	-	-	-	-	-	-	-	-	-	-	-	-	-
Cuphophyllus virgineus (Wulfen) Kovalenko	-	-	-	-	-	-	12	27	-	-	-	-	2	-	28	-	-	-	-	-	14	-	10	3	-	-	-	-
Cyathus olla (Batsch) Pers.	-	2	11	21	-	7	17	5	-	-	-	-	-	7	-	-	-	-	-	-	-	-	-	-	-	-	-	-
Cyathus stercoreus (Schwein.) de Toni	-	-	-	-	-	10	-	-	-	-	-	-	-	-	-	-	-	-	-	-	-	-	-	-	-	-	-	-
Deconica coprophila (Bull.) P. Karst.	-	-	-	-	-	-	4	-	-	-	-	4	-	-	-	-	-	-	-	-	-	-	-	-	-	-	-	-

Deconica inquilina (Fr.) Pat. ex Romagn.	-	-	-	-	-	-	-	3	-	-	-	-	-	-	4	-	-	-	-	-	-	-	-	-	-	-	-	-
Deconica montana (Pers.) P.D. Orton	-	-	2	-	-	-	-	-	-	-	-	-	-	-	-	-	-	-	-	-	-	-	2	-	-	-	-	-
Disciseda bovista (Klotzsch) Henn.	-	2	1	2	-	1	14	-	-	-	-	-	-	-	-	-	-	-	-	-	-	-	1	-	-	-	-	-
Disciseda candida (Schwein.) Lloyd	-	11	18	5	-	4	6	1	-	-	-	-	-	-	-	-	-	-	-	-	-	-	-	-	-	-	-	-
Disciseda verrucosa G. Cunn.	-	-	5	1	-	-	8	13	-	-	-	-	-	-	-	-	-	-	-	-	-	-	-	-	-	-	-	-
Entoloma conferendum (Britzelm.) Noordel.	-	-	-	-	-	-	-	-	-	-	-	-	-	1	-	-	-	-	-	-	-	-	-	-	-	1	-	-
Entoloma incanum (Fr.) Hesler	-	-	-	-	-	-	-	-	-	-	-	-	-	-	-	-	-	-	-	-	-	-	-	6	-	-	-	-
Entoloma sericeum Quél.	-	-	-	1	-	-	-	1	-	-	-	-	-	-	-	-	-	-	-	-	-	-	-	4	-	-	-	-
Entoloma serrulatum (Fr.) Hesler	-	-	-	1	-	-	-	-	-	-	-	-	-	-	-	-	-	-	-	-	-	-	-	-	-	-	-	-
Flammulina ononidis Arnolds	-	-	-	-	-	-	-	-	-	-	-	-	-	-	-	-	-	-	95	50	-	-	-	-	-	-	-	-
Galerina embolus (Fr.) P. D. Orton	-	-	4	-	-	-	-	-	-	-	-	-	-	-	-	-	-	-	-	-	-	-	-	-	-	-	-	-
Galerina graminea (Velen.) Kühner	4	-	3	18	-	-	77	6	-	-	6	-	4	-	27	-	-	-	-	-	-	-	-	-	-	-	-	-
Galerina marginata (Batsch) Kühner	-	-	-	-	-	-	-	-	-	-	-	-	-	-	-	-	-	-	-	-	5	-	-	-	-	-	-	-
Galerina pumila (Pers.) Singer	-	-	-	-	-	2	-	-	-	-	-	-	-	-	-	-	-	-	-	-	4	3	-	-	-	-	-	-
Galerina triscopa (Fr.) Kühner	-	-	-	-	-	-	-	-	-	-	-	-	-	-	-	-	-	-	-	-	-	-	2	-	-	-	-	-
Gastrosporium simplex Mattir	3	8	7	2	5	23	173	7	-	-	-	-	-	-	-	-	-	-	-	-	-	7	34	4	-	-	-	-
Geastrum berkeleyi Massee	-	-	-	5	-	-	29	-	-	-	-	-	-	-	-	-	-	-	-	-	-	-	-	-	-	-	-	-
Geastrum campestre Morgan	-	24	69	44	-	-	25	-	-	-	-	-	-	-	-	-	-	-	-	-	-	5	-	-	-	-	-	-
Geastrum kotlabae V. J. Stanek	-	7	-	-	-	-	-	-	-	-	-	-	-	-	-	-	-	-	-	-	-	-	-	-	-	-	-	-
Geastrum minimum Schwein.	-	26	29	6	2	27	132	6	-	-	-	-	-	-	-	-	-	-	-	-	-	-	-	-	-	-	-	-
Geastrum pectinatum Pers.	-	15	14	13	-	-	8	-	-	-	-	-	-	-	-	-	-	-	-	-	-	-	-	-	-	-	-	-
Geastrum striatum DC.	-	51	100	60	-	9	76	2	-	-	-	-	-	1	-	-	-	-	-	-	-	3	6	6	-	-	-	-
Geopora arenicola (Lév.) Kers	-	-	-	-	-	-	-	2	3	-	-	-	-	-	-	-	-	-	-	-	-	-	-	-	-	-	-	-

Gliophorus laetus (Pers.) Herink	-	-	-	-	-	-	10	7	-	-	-	-	-	-	-	-	-	-	-	-	-	-	-	-	-	-	-	-
Gymnopus ocior (Pers.) Antonín & Noordel.	-	-	-	-	-	-	-	-	2	-	-	-	-	-	-	-	-	-	-	-	-	-	-	-	-	-	-	-
Hebeloma mesophaeum (Pers.) Quél.	-	-	-	-	-	-	-	-	-	-	-	-	-	-	-	-	-	-	-	-	-	-	-	3	-	-	-	-
Hemimycena crispata (Kühner) Singer	-	6	-	-	-	-	-	-	-	-	-	-	-	-	-	-	-	-	-	-	-	-	-	-	-	-	-	-
Hemimycena cucullata (Pers.) Singer	-	-	-	-	-	-	-	-	-	-	-	-	-	-	5	-	-	-	-	-	-	-	-	-	-	-	-	-
Hemimycena mairei (E.-J. Gilbert) Singer	-	-	-	-	-	-	3	-	-	-	-	-	-	-	-	-	-	-	-	-	-	-	-	-	-	-	-	-
Hygrocybe acutoconica (Clem.) Singer	-	-	-	-	-	-	-	-	-	-	-	-	-	-	-	-	-	-	-	-	-	7	-	-	-	-	-	-
Hygrocybe ingrata J.P. Jensen & F.H. Møller	-	-	-	-	-	-	-	-	-	-	-	-	-	-	-	-	-	-	-	-	-	-	1	-	-	-	-	-
Hygrocybe mucronella (Fr.) P. Karst.	-	-	-	-	-	-	-	-	-	-	-	-	-	-	-	-	-	-	-	-	6	-	-	-	-	-	-	-
Hygrocybe parvula (Peck) Murrill	-	-	-	-	-	-	-	-	-	-	-	-	-	-	-	-	-	-	-	-	2	-	-	-	-	-	-	-
Hymenoscyphus repandus (W. Phillips) Dennis	-	-	-	-	-	-	-	-	-	-	-	-	-	-	-	-	-	-	-	-	-	-	-	-	-	-	-	20
Hymenoscyphus scutula (Pers.) W. Phillips	-	-	-	-	-	-	-	-	-	-	-	-	-	8	-	-	-	-	-	-	-	-	-	-	-	-	-	-
Hypholoma fasciculare (Huds.) P. Kumm.	-	-	-	-	-	-	-	-	-	-	-	-	-	-	-	-	-	-	-	-	2	-	-	-	-	-	-	-
Inocybe dulcamara (Pers.) P. Kumm.	-	-	-	-	-	-	-	-	-	-	-	-	-	-	-	-	2	-	-	-	-	-	-	-	-	-	-	-
Inocybe serotina Peck	3	-	-	-	-	-	-	-	-	-	-	-	-	-	-	-	-	-	-	-	-	-	-	-	-	-	-	-
Lactarius torminosus (Schaeff.) Pers.	-	-	-	-	-	-	-	-	-	-	-	-	-	-	-	-	-	-	-	-	-	14	13	5	-	-	-	-
Lentinus brumalis (Pers.) Zmitr.	-	-	-	-	-	-	-	-	-	-	-	-	-	-	-	-	-	-	-	-	-	-	-	2	-	-	-	-
Lepiota cristata (Bolton) P. Kumm.	1	4	4	5	-	-	-	3	-	-	-	-	-	-	-	-	-	-	-	-	-	-	-	-	-	-	-	-
Lepiota erminea (Fr.) P. Kumm.	-	-	3	2	-	-	3	-	-	-	-	-	-	-	-	-	-	-	-	-	-	-	-	-	-	-	-	-
Lepiota oreadiformis Velen.	1	-	-	-	-	-	-	-	-	-	-	-	-	-	-	-	-	-	-	-	-	-	-	-	-	-	-	-
Lepista luscina (Fr.) Singer	-	-	-	-	-	-	-	-	-	-	-	-	-	-	-	8	-	-	-	-	-	-	-	-	-	-	2	-

Lepista personata (Fr.) Cooke	-	-	-	-	-	-	5	-	-	-	-	-	-	-	-	-	-	-	-	-	-	-	-	-	-	-	-	-
Lepista sordida (Schumach.) Singer	-	-	-	-	-	-	6	-	-	-	-	-	-	-	-	-	-	-	-	-	-	-	-	-	-	-	-	-
Leucocoprinus cretaceus (Bull.) Locq.	-	-	-	-	-	-	-	-	-	-	-	-	2	-	1	-	-	-	-	-	-	-	-	-	-	-	-	-
Lichenomphalia umbellifera (L.) Redhead, Lutzoni, Moncalvo & Vilgalys	-	-	2	-	5	-	-	-	8	-	-	-	-	-	-	-	-	-	-	-	2	-	-	-	-	-	-	-
Lycoperdon dermoxanthum Vittad.	5	3	23	-	-	1	3	-	-	-	-	-	-	-	-	-	-	-	-	-	-	-	-	-	-	-	-	-
Lycoperdon excipuliforme (Scop.) Pers.	-	-	-	-	6	1	-	-	-	-	-	-	-	1	-	-	-	-	-	-	-	-	-	-	-	-	-	-
Lycoperdon lividum Pers.	-	-	17	30	3	6	10	5	-	-	-	-	-	-	2	-	-	-	-	-	-	-	-	-	-	-	-	-
Lycoperdon molle Pers.	-	6	-	-	-	-	-	-	-	-	-	-	-	-	-	-	-	-	-	-	-	-	-	-	-	-	-	-
Lycoperdon perlatum Pers.	-	-	23	-	-	-	-	-	-	-	-	-	-	-	-	-	-	-	-	-	-	-	3	-	-	-	-	-
Lycoperdon pratense Pers.	-	-	3	-	-	1	20	4	-	-	-	-	-	1	-	-	-	-	-	-	-	-	-	-	-	-	-	-
Macrolepiota excoriata (Schaeff.) Wasser	-	-	-	-	-	-	6	2	-	-	-	-	-	-	-	3	-	-	-	-	-	-	-	-	-	-	-	-
Macrolepiota mastoidea (Fr.) Singer	-	-	1	-	-	-	8	-	-	-	12	-	-	-	5	-	-	-	-	-	-	-	-	-	-	-	-	-
Macrolepiota procera (Scop.) Singer	-	-	-	-	-	-	-	-	-	-	-	-	-	-	-	-	-	-	-	-	-	-	1	-	-	-	-	-
Macrolepiota rubescens (L.M. Dufour) Pázmány	-	-	-	-	-	-	-	-	-	-	-	-	-	-	2	-	-	-	-	-	-	-	-	-	-	-	-	-
Macrolepiota venenata Bon	-	-	2	-	-	-	-	-	-	-	-	-	-	-	-	-	-	-	-	-	-	-	-	-	-	-	-	-
Marasmius curreyi Berk. & Broome	-	-	-	-	-	-	-	-	-	-	-	-	-	2	-	-	-	-	-	-	-	-	-	-	-	-	-	-
Marasmius limosus Quél.	-	-	-	-	-	-	-	-	-	-	-	-	-	121	-	-	-	-	-	-	-	-	-	-	-	-	-	-
Marasmius oreades (Bolton) Fr.	-	-	1	12	3	10	73	6	-	12	11	8	-	3	3	-	-	-	-	-	-	8	-	-	-	-	-	-
Marasmius rotula (Scop.) Fr.	-	-	-	-	-	-	-	3	-	-	-	-	-	-	-	-	-	-	-	-	-	-	-	-	-	-	-	-
Melanoleuca melaleuca (Pers.) Murrill	-	-	-	-	-	-	-	-	-	-	-	-	1	-	-	-	-	-	-	-	-	-	-	-	-	-	-	-
Mycena aetites (Fr.) Quél.	-	-	7	5	-	-	6	8	-	-	-	-	-	-	2	14	-	-	-	-	-	-	-	-	-	-	-	-

Mycena epipterygia (Scop.) Gray	-	-	-	-	-	-	4	-	-	-	-	-	-	-	-	-	-	-	-	-	-	-	-	-	-	-	-	-
Mycena galericulata (Scop.) Gray	-	-	-	-	-	-	4	-	-	-	5	-	-	-	-	-	-	-	-	-	-	-	-	-	-	-	-	-
Mycena galopus (Pers.) P. Kumm. var. nigra Rea	-	-	4	-	-	-	5	-	-	-	-	-	-	-	-	-	-	-	-	-	-	-	-	-	-	-	-	-
Mycena leptocephala (Pers.) Gillet	-	-	-	-	-	-	7	-	-	-	-	-	-	-	-	-	-	-	-	-	-	-	-	-	-	-	-	-
Mycena olivaceomarginata (Massee) Massee	-	-	48	-	-	-	2	-	-	-	-	-	-	-	-	-	-	-	-	-	-	-	18	-	-	-	-	-
Mycena pseudopicta (J. E. Lange) Kühner	-	-	49	5	-	-	130	11	-	-	52	-	3	-	52	-	-	-	5	4	-	-	-	-	-	-	9	-
Omphalina pyxidata (Bull.) Quél.	-	-	-	10	-	-	-	-	-	-	-	-	-	-	-	-	-	-	-	-	-	-	-	-	-	-	-	-
Orbilia sarraziniana Boud.	-	-	-	-	-	-	-	-	-	-	-	-	-	-	-	-	-	-	182	-	-	32	-	-	-	-	-	-
Panaeolina foenisecii (Pers.) Maire	-	-	-	-	-	-	-	-	-	-	-	-	-	-	-	-	3	-	4	-	-	-	-	-	-	-	-	-
Panaeolus olivaceus F. H. Møller	-	-	-	-	-	-	-	-	-	6	-	-	-	-	-	-	-	-	-	-	-	-	-	-	-	-	-	-
Panaeolus papilionaceus (Bull.) Quél.	-	-	-	-	-	-	-	2	-	-	-	-	1	-	10	-	-	-	-	-	-	1	1	1	-	-	-	-
Panus conchatus (Bull.) Fr.	-	-	-	-	-	-	-	-	-	-	-	-	-	-	-	-	-	-	-	-	-	-	3	-	-	-	-	-
Panus rudis Fr.	-	-	-	1	-	-	-	-	-	-	-	-	-	-	-	-	-	-	-	-	-	-	-	-	-	-	-	-
Peziza vesiculosa Bull.	-	-	-	-	1	-	-	-	2	-	-	-	-	-	-	-	-	-	-	-	-	-	-	4	-	-	-	-
Phaeoclavulina abietina (Pers.) Giachini	-	-	-	-	-	-	-	-	-	-	2	-	-	-	-	-	-	-	-	-	-	-	-	-	-	-	-	-
Phellinus pomaceus (Pers.) Maire	-	-	-	-	-	-	-	-	1	-	-	-	-	-	-	-	-	-	-	-	-	-	-	-	-	-	-	-
Picipes badius (Pers.) Zmitr. & Kovalenko	-	-	-	-	-	-	-	-	-	-	-	-	-	-	-	-	-	-	-	-	-	-	-	2	-	-	-	-
Protostropharia semiglobata (Batsch) Redhead, Moncalvo & Vilgalys	-	-	-	-	-	-	-	-	-	-	15	-	-	-	-	-	-	-	-	-	-	-	-	-	-	-	-	-
Psathyrella corrugis (Pers.) Konrad & Maubl.	-	-	-	-	-	-	-	-	-	-	-	-	-	-	-	-	-	-	-	-	-	-	6	-	-	-	-	-
Psathyrella fatua (Fr.) Konrad & Maubl.	-	-	-	1	-	-	-	-	-	-	-	-	-	-	-	-	-	-	-	-	-	-	-	-	-	-	-	-
Psathyrella prona (Fr.) Gillet	-	-	-	-	-	-	-	-	-	-	-	-	-	-	-	-	-	-	-	-	-	-	8	-	-	-	-	-

Psathyrella pseudogracilis (Romagn.) M. M. Moser	-	-	-	-	-	-	-	-	-	-	-	-	-	-	-	-	-	-	-	-	-	7	-	-	-	-	-	-
Psathyrella spadiceogrisea (Schaeff.) Maire	-	-	-	-	2	-	-	-	-	-	-	-	-	-	-	-	-	-	-	-	-	-	-	-	-	-	-	-
Psilocybe coronilla (Bull.) Noordel.	-	-	2	-	-	-	5	-	-	-	-	-	-	-	-	-	-	-	-	-	-	-	-	-	-	-	-	-
Rhodocybe parilis (Fr.) Singer	-	-	-	-	-	-	1	-	-	-	-	-	-	-	-	-	-	-	-	-	-	-	-	-	-	-	-	-
Rickenella fibula (Bull.) Raithelh.	-	-	-	-	-	-	-	-	3	-	-	-	-	-	-	-	-	-	-	-	-	-	-	-	-	-	-	-
Schizophyllum commune Fr.	-	-	-	-	-	-	4	-	54	-	-	-	-	-	-	-	-	-	-	-	-	-	11	-	-	-	-	-
Sclerogaster gastrosporioides Pilát & Svrček	-	-	-	-	-	-	-	-	-	-	-	-	-	-	-	-	-	-	-	-	-	-	3	-	-	-	-	-
Stereum hirsutum (Willd.) Pers.	-	-	-	-	-	-	-	-	-	-	-	-	-	-	-	-	-	-	-	-	5	-	14	-	-	-	-	-
Stropharia albonitens (Fr.) Quél.	-	-	2	-	-	-	-	-	-	-	-	-	-	-	-	-	-	-	-	-	-	-	-	-	-	-	-	-
Tephrocybe ambusta (Fr.) Donk	-	-	-	-	-	-	-	-	1	-	-	-	-	-	-	-	-	-	-	-	-	-	-	-	-	-	-	-
Trametes hirsuta (Wulfen) Lloyd	-	-	-	-	-	-	-	-	-	-	-	-	-	-	-	-	-	-	-	-	4	-	5	-	-	-	-	-
Trametes ochracea (Pers.) Gilb. & Ryvarden	-	-	-	-	-	-	20	-	-	-	-	-	-	-	-	-	-	-	-	-	-	-	-	-	-	-	-	-
Tricholoma pessundatum (Fr.) Quél.	-	-	-	-	-	-	-	-	-	-	-	-	-	-	-	-	-	-	-	-	-	-	-	6	-	-	-	-
Tricholoma portentosum (Fr.) Quél.	-	-	-	-	-	-	-	-	-	-	-	-	-	-	-	-	-	-	-	-	-	-	2	-	-	-	-	-
Tubaria conspersa (Pers.) Fayod	-	-	4	3	-	-	-	-	-	-	-	-	-	-	6	-	-	-	-	-	-	-	-	-	-	-	-	-
Tubaria furfuracea (Pers.) Gillet	-	-	-	-	-	-	17	-	-	-	-	-	-	-	-	-	-	-	-	-	-	-	-	-	-	-	-	-
Tulostoma brumale Pers.	8	-	-	4	-	-	-	-	-	-	-	-	-	-	-	-	-	-	-	-	-	-	-	-	-	-	-	-
Tulostoma brumale var brumale Pers.	-	51	62	10	7	-	69	4	5	-	-	60	-	-	-	-	-	-	-	-	-	1	8	-	-	-	-	-
Tulostoma kotlabae Pouzar	-	2	2	1	1	-	-	-	1	6	-	-	-	-	-	-	-	-	-	-	-	1	1	-	-	-	-	-
Tulostoma melanocyclum Bres.	4	84	23	13	7	7	103	3	-	-	3	-	-	7	-	-	-	-	-	-	1	-	-	-	-	-	-	-
Tulostoma pallidum Lloyd	-	-	4	-	-	-	-	3	3	-	-	-	-	-	-	-	-	-	-	-	-	-	2	1	-	-	-	-

Tulostoma squamosum (J. F. Gmel.) Pers.	4	151	225	28	4	21	38	6	-	22	-	-	-	-	-	-	-	-	-	-	-	-	2	2	-	-	-	-
Xylaria hypoxylon (L.) Grev.	-	-	-	-	-	-	-	-	-	-	-	-	-	-	-	-	-	-	-	-	-	1	-	-	-	-	-	-
